# Quantitative analysis of polycomb response elements (PREs) at identical genomic locations distinguishes contributions of PRE sequence and genomic environment

**DOI:** 10.1186/1756-8935-4-4

**Published:** 2011-03-16

**Authors:** Helena Okulski, Birgit Druck, Sheetal Bhalerao, Leonie Ringrose

**Affiliations:** 1 IMBA, Institute of Molecular Biotechnology GmBH, Dr. Bohr-Gasse 3, 1030 Vienna, Austria; 2 Roche Austria GmBH, Clinical Operations, Engelhorngasse 3, 1211 Vienna, Austria; 3 Department of Neurobiology, Stanford University, Stanford, CA 94305, USA

## Abstract

**Background:**

Polycomb/Trithorax response elements (PREs) are *cis*-regulatory elements essential for the regulation of several hundred developmentally important genes. However, the precise sequence requirements for PRE function are not fully understood, and it is also unclear whether these elements all function in a similar manner. *Drosophila *PRE reporter assays typically rely on random integration by P-element insertion, but PREs are extremely sensitive to genomic position.

**Results:**

We adapted the ΦC31 site-specific integration tool to enable systematic quantitative comparison of PREs and sequence variants at identical genomic locations. In this adaptation, a *miniwhite *(*mw*) reporter in combination with eye-pigment analysis gives a quantitative readout of PRE function. We compared the Hox PRE *Frontabdominal-7 *(*Fab-7*) with a PRE from the *vestigial *(*vg*) gene at four landing sites. The analysis revealed that the *Fab-7 *and *vg *PREs have fundamentally different properties, both in terms of their interaction with the genomic environment at each site and their inherent silencing abilities. Furthermore, we used the ΦC31 tool to examine the effect of deletions and mutations in the *vg *PRE, identifying a 106 bp region containing a previously predicted motif (GTGT) that is essential for silencing.

**Conclusions:**

This analysis showed that different PREs have quantifiably different properties, and that changes in as few as four base pairs have profound effects on PRE function, thus illustrating the power and sensitivity of ΦC31 site-specific integration as a tool for the rapid and quantitative dissection of elements of PRE design.

## Background

Polycomb/Trithorax response elements (PREs) are *cis*-regulatory DNA elements that recruit both the Polycomb group (PcG) and Trithorax group (TrxG) proteins, required respectively for gene silencing and activation [1-3]. PREs were first identified in the homeotic gene clusters of the Bithorax complex (BX-C) [4-6] and the Antennapedia complex (ANT-C) [7] in *Drosophila*. Genomewide studies in flies and vertebrates have since identified several hundred additional PcG target genes involved in cell-fate specification, cell signaling and proliferation [8-18]. However, functional studies of PRE elements themselves have been performed for only a few of these loci. These studies, based on transgenic reporter assays, have shown that several *Drosophila *PREs share common molecular and genetic features. These include recruitment of PcG and TrxG proteins to an ectopic site, pairing-sensitive silencing (PSS) and variegation of a linked reporter gene, and genetic dependence on the PcG and TrxG proteins [4-7,19,20]. Furthermore, several PREs have been shown to act as epigenetic memory elements, conferring mitotic and in some cases meiotic inheritance of the previously established silenced or activated transcriptional states of their associated reporter genes [4-6,21-25]. Recently, two examples of mammalian PREs that share some of these features were reported [26,27].

The DNA sequence requirements for PRE function are only partially understood. In flies, several DNA motifs have been shown to play an essential role in PcG and TrxG recruitment and in gene silencing or activation at *Drosophila *PREs. These include binding sites for the PcG proteins Pleiohomeotic (PHO) and PHO-like (PHOL) [28-30], the Zeste protein (Z) [31,32] and the GAGA factor/Trithorax-like (GAF/TRL) [33], which binds a similar motif to that recognized by Pipsqueak (PSQ) [34,35]. Clusters of pairs of these motifs are sufficient for computational detection of a subset of known *Drosophila *PREs and for the prediction of further PREs, many of which have been confirmed experimentally [8,36]. However these motifs alone are not sufficient to predict all PREs in the genome, nor to fulfill PRE function [37], and functional roles for additional DNA sequence motifs have been defined for specific PREs, including binding sites for Dorsal switch protein (DSP)1 [37], Grainyhead (GRH) [38], the SP1/Kruppel-like factor (KLF) family of transcription factors [39] and several other unidentified proteins [1,2,25]. In addition, with the use of sequence mining, further DNA motifs have been found to be enriched at PRE elements [8,11], and have thus been proposed to play a role in PRE regulation, but their function has not been tested experimentally.

Interestingly, for the functional motifs defined to date, different PREs have different combinations of motifs, with no detectable preferred order or number [8]. Furthermore the order and number of these motifs at PREs is varies greatly between *Drosophila *species, even within orthologous PREs [36]. These observations raise the question of whether these differences in sequence simply reflect redundancy of design, or whether they are in fact important for determining different functional outputs of different PREs. Several studies support the idea that PREs from different genes are functionally similar to each other despite differences in sequence. For example, transgenic reporter assays in which PREs are linked to a heterologous enhancer have shown that PREs from different Hox genes and from the *engrailed *gene (*en*), can maintain reporter-gene expression in the pattern previously determined by the enhancer, showing that these PREs are interchangeable in this maintenance assay [4,6,25]. A recent study used gene conversion to examine the effect of exchanging PRE sequences within the BX-C. A 185 bp core sequence of the *bxd *(*bithoraxoid*) PRE was replaced within the endogenous *Ubx *(*Ultrabithorax*) locus by equivalent core sequences of two PREs from the *Abd-B *(*Abdominal-B*) gene. Core sequences were defined as minimal fragments that contain known functional motifs and have PRE activity in reporter assays. The two core PREs tested gave full genetic rescue of the *bxd *deletion phenotype, again suggesting interchangeability between PRE cores [40].

However there are also results indicating functional differences between PREs from different genes. Genetic experiments have shown that the *Ubx *and *AbdB *genes respond differently to the removal of PcG proteins upon induction of PcG mutant clones of cells, suggesting different strengths of silencing mediated by the PREs at these loci [41]. For other PREs outside the Hox complexes, several different functions have been documented. For example, the PREs of the *polyhomeotic *gene (*ph*) maintain an equilibrium between activation and silencing instead of an on and off switch as proposed for the Hox PREs [42,43]. The *hedgehog *PRE (*hh*) has been shown to switch several times during development [24]. A PRE at the *Cyclin *(*Cyc*)*A *gene mediates PcG-dependent regulation of dynamic *CycA *expression during development [44]. These studies indicate that PREs from several different genes have different properties. However, relatively few studies have compared different PREs in the same experimental setup. Furthermore, with the exception of two recent gene-conversion studies reported previously [40,45], most *Drosophila *PRE studies have relied on transgenic assays using random P-element insertion. This approach has the advantage that it is more rapid than gene conversion, but the disadvantage that PREs are subject to genomic position effects [4,5,19]. Thus, to gain a quantitative understanding of differences in PRE function, and to determine the contribution of specific DNA sequences, it is essential to compare PREs, sequence variants and control constructs at the same genomic locus.

We report a quantitative comparison of PREs in *Drosophila *using the site-specific ΦC31 integration tool [46]. We compared the Hox PRE *Frontabdominal-7 *(*Fab-7*) from the *Abd-B *gene with a PRE from the *vestigial *(*vg*) locus. To distinguish between the effects of PRE sequence and of genomic environment, we generated transgenic reporter fly lines carrying each PRE at four different, precisely characterized, landing sites. This analysis demonstrates that the *Fab-7 *and *vg *PREs do indeed have inherently different properties, in terms of their ability to silence the reporter gene, the extent of pairing sensitivity and their interaction with the genomic environment. For the *vg *PRE, we present a mutational analysis, identifying an essential function in silencing for a motif (GTGT) that was previously discovered by bioinformatic sequence mining to be enriched at many PRE sites [8]. In summary, this study gives quantitative insights into the similarities and differences in PREs from distinct genes, and identifies DNA sequences essential for silencing. Thus, site-specific ΦC31 integration offers a powerful approach for the rapid and quantitative dissection of elements of PRE design.

## Results

### Site-specific integration enables analysis of PREs at known genomic locations

**Figure 1:**
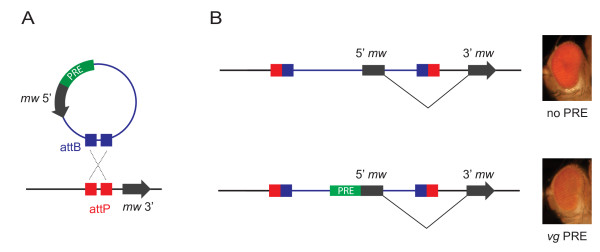
**Comparison of Polycomb response elements (PREs) by ΦC31 site-specific integration**. **(A) **The plasmid pKC27 carries the PRE of interest, the 5' end of the *miniwhite *(*mw*) reporter gene including the first exon and 195 bp of the first intron and the phage ФC31 attB recognition site. The genomic landing site carries an attP site followed by a genomic DNA fragment of the *white *gene containing the last 205 bp of intron 1 and the remaining downstream exons. **(B) **Correct site-specific integration is detected by the expression of a functional *mw *reporter gene. (Top right) flies carrying only the *mw *reporter have red eyes. (Bottom right) The *mw *reporter is repressed in the presence of a PRE, resulting in a decrease in eye coloration.

To enable quantitative comparison of PREs and their variants, and to avoid the problem of genomic position effects, we adapted the ΦC31 site-specific integration system to make it suitable for comparative studies of PREs at the same genomic location (Figure 1) [46]. To this end we used an adaptation of the 'split *white*' system [47], in which a functional *white *gene (*w*) is reconstituted only upon the correct site-specific integration event. We replaced the *white *gene with *miniwhite *(*mw*; FBtp0000155). The *mw *gene contains the coding sequences of the *white *gene, but the 5" regulatory sequences are reduced to 300 bp. The *mw *gene is typically used as a sensitive reporter gene for PRE effects [5], and serves here both as a transgenic marker and as a reporter of PRE activity.

Landing-site fly lines were generated by standard P-element integration into *yw *flies, using *yellow *(*y*) as a transformation marker, and their genomic locations were mapped (Table 1). The landing site carries an attP site followed by a genomic DNA fragment of the *white *gene containing the last 205 bp of intron 1, and the remaining downstream exons. The landing-site lines used here for comparative studies of PREs were selected by means of two criteria: no Polycomb protein binding within a window of ±5 kb from the landing site [9,11], and an intergenic location (Table 1). The donor plasmid, pKC27_*mw*, carries the *mw *promoter, followed by exon 1, the first 195 bp of intron 1, and an attB site (Figure 1A). Upon site-specific integration of pKC27_*mw *into the landing site, a functional *mw *gene is reconstituted with the attB/attP site in the intron (Figure 1B). PREs were cloned into the pKC27_*mw *plasmid directly upstream of the *mw *reporter gene (Figure 1A). Transgenics were generated (described in Methods), by co-injection of landing-site lines with pKC27_*mw *derivatives and a helper plasmid encoding the ΦC31 integrase. Flies carrying only the *mw *reporter had red eyes (Figure 1B). In the presence of a PRE, the eye color gives a readout of the PRE activity at the same landing site. Typically, addition of a PRE sequence led to downregulation of *mw *(Figure 1B). In summary, site-specific integration combined with an *mw *reporter enables comparison of PREs and control constructs at the same genomic loci.

**Table 1:** Characteristics of landing sites^1^

Site	**Cytological location**^**2**^	**Genomic location**^**2**^	**Gene density**^**3**^	**Adjacent genes**^**4**^	**Next PC binding sites**^**5**^	**Chromatin type**^**6**^	**GTGTG**^**8**^	**GAGAG**^**8**^	**GCCAT**^**8**^
1	chr. 2L, 38E3	20,716,266	2	+8 kb: *CG9316*	+42 kb: *dia*	Red	59	36	51
								
				-1.7 kb: *Hr38*	-947 kb: *bsh*				

2	chr. 2R, 46E1	5,965,083	4	+0.8 kb: *egr*	+1396 kb: *inv*	Red	47	25	53
								
				-1.9 kb: *CG1371*	-97 kb: *eve*				

3	chr. 2R, 58F4	18,549,410	12	+29 bp: *CG42566*	+205 kb: *fd59A*	Blue	43	26	36
								
				-0.5 kb: *CG42565*	-375 kb: *dve*				

4	chr. 3R, 100E3	27,899,491	1	Telomeric region	-	Yellow	41	38	17
								
				-5.3 kb: *Map205*	-5.3 kb: *Map205*				

^1^All four landing sites are located in intergenic regions with no PcG binding within a window or ±5 kb [9,11].

^2^Cytological and genomic locations are shown according to FlyBase version FB2010_05.

^3^Gene density: number of genes whose gene span overlaps a ±10 kb window from the landing site.

^4^Adjacent genes: distance between genomic coordinate of the landing site and that of the closest edge of the next adjacent gene span in both directions (- = upstream, + = downstream according to genomic coordinates). Note that site 3 is located in the telomeric region of chromosome 3R, thus there is no downstream gene.

^5^Next PC binding sites: the closest Polycomb binding site up and downstream of the landing site was identified according to [9,11].

^6^Chromatin type: Drosophila chromatin type according to [66] in which each landing site is located. 'Red' and 'yellow' chromatin types share features of transcriptionally active euchromatin but differ in the presence of specific factors (see Discussion for details). Note that the border of 'yellow' chromatin extends to 1.7kb upstream of site 4. This site is not assigned to a chromatin type in [66]. 'Blue' chromatin is enriched for PcG proteins and H3K27me3.

^8^GTGTG, GAGAG, GCCAT: the number of occurrences of each of these motifs within 10kb up and downstream of each landing site is shown.

### A 1.6 kb fragment of the *vestigial *PRE is sufficient to mediate Polycomb-dependent silencing

The site-specific integration tool enables not only the comparison of PREs and control constructs at the same genomic location, but also the comparison of different PREs. We chose to compare the well-characterized *Fab-7 *PRE from the BX-C with a less well-characterized PRE from outside the Hox complexes, namely, from the *vestigial (vg) *gene. The *vg *gene is essential for correct cell-fate specification in wing and haltere development [48] and during muscle differentiation [49,50], and is also expressed in the embryonic central nervous system [48,51].

**Figure 2:**
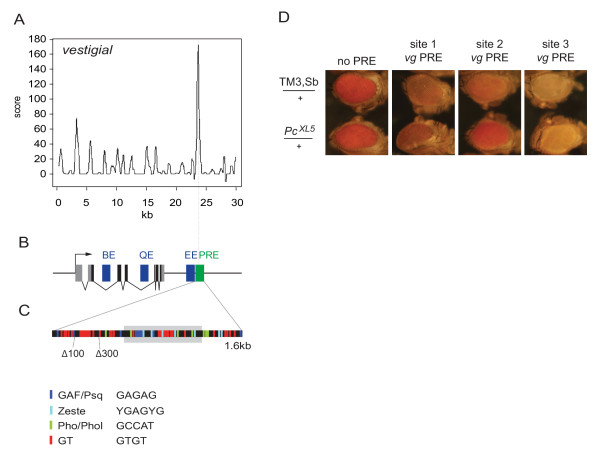
**Functional analysis of a 1.6 kb fragment of the *vg *Polycomb response element (PRE)**. **(A) **Score plot showing PRE prediction at the *vestigial (vg) *locus according to Ringrose *et al. *[8]. **(B) ***vg *locus, showing coding exons (black,) non-coding exons (grey), enhancers, (blue) and downstream PRE (green). BE = boundary enhancer [73,74]; EE = predicted embryonic muscle enhancer [75]; QE = quadrant enhancer [73,74]. **(C) **DNA motifs in a *vg *PRE fragment of 1.6 kb. The grey box corresponds to the highest-scoring region shown in (A). ZESTE, GAGA factor/Pipsqueak (GAF/PSQ) and Pleiohomeotic/Pleihomeotic-like (PHO/PHOL) DNA-binding motifs are shown. In addition, the GTGT motif, found to be enriched in many PREs [8,11] is enriched in the high scoring region and sequences flanking this region. **(D) **The 1.6 kb *vg *PRE shows responsiveness to Polycomb. Eye colors of 5-day-old adult male flies are shown. Flies are homozygous for transgenes on chromosome II as indicated above each panel, and heterozygous on chromosome III for either a balancer chromosome (top; *TM3,Sb/+*) or the *Pc*^*XL5 *^mutation (bottom;*Pc*^*XL5*^*/+*). The presence of the *Pc*^*XL5 *^mutation had no effect on the control construct lacking the PRE at site 1 (no PRE, left) or sites 2 and 3 (not shown), but led to a loss of *mw *repression in flies carrying the *vg *PRE at all three sites.

The *Fab-7 *PRE regulates the homeotic gene *Abd-B*, and is located downstream of the gene, approximately 70 kb from the promoter [52,53]. Similarly, the *vg *PRE that we studied is located downstream of the gene, approximately 20 kb from the promoter (Figure 2A,B). This *vg *PRE was identified by bioinformatic prediction and confirmed as a PcG binding site by chromatin immunoprecipitation (ChIP) [8] (Figure 2A). A 3 kb fragment containing the highest-scoring region shown in Figure 2A has previously been verified as a PRE in transgenic reporter assays [54]. Later genomewide profiling studies showed that both this *vg *PRE and the *vg *promoter are enriched for PcG and/or TrxG proteins in *Drosophila *cell culture [9,10,16], in embryos [11,55] and in larval imaginal discs [55]. The promoter site also contains GAF, ZESTE and PHO binding sites (observable by the peak at this site in the score plot on Figure 2A), but has not been verified as a PRE in transgenic assays. Thus, we focused our analysis on the downstream *vg *PRE.

Comparison of this *vg *PRE with the *Fab-7 *PRE in terms of the occurrence of DNA-binding motifs identified a distinct number, density and distribution of motifs (Figure 2C; see Additional file 1, Figure S1). Transgenic studies of the *Fab-7 *PRE identified a minimal 219 bp fragment that is sufficient for PRE activity [56], (see Additional file 1, Figure S1). This fragment falls within the highest-scoring region in computational predictions, based on the density of pairs of GAF, ZESTE and PHO binding sites [8]. However the sequences flanking this fragment contain additional binding sites for these proteins, thus we cloned 1.6 kb of the *Fab-7 *PRE sequence, centered on the minimal PRE (see Additional file 1, Figure S1). The minimal functional fragment of the *vg *PRE has not been defined, but the site of highest motif density is identifiable as the highest-scoring region in the study by Ringrose *et al. *[8] (Figure 2C, grey box), thus to enable comparisons with the *Fab-7 *PRE, we cloned 1.6 kb of the *vg *PRE centered on this site. This 1.6 kb fragment falls within the 3 kb fragment previously shown to fulfill PRE function [54].

**Figure 3:**
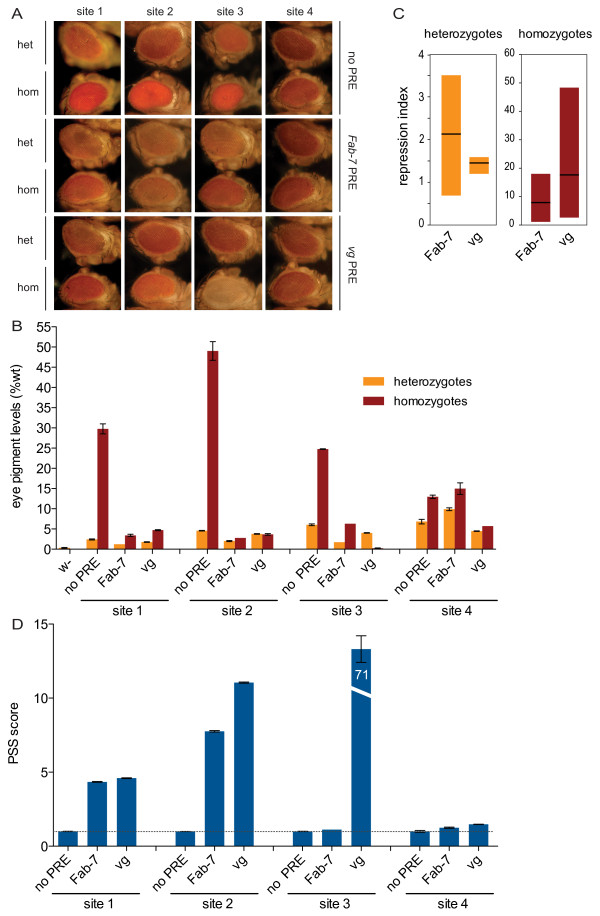
**Comparison of *Fab-7 *and *vg *Polycomb response elements (PREs) at four landing sites**. **(A) **Eye phenotype of transgenic fly lines; 5-day-old male flies are shown. In each row, flies (top) heterozygous and (bottom) homozygous for each transgene are shown at all four landing sites. (Top row) No PRE: *mw *reporter alone; (middle row) *Fab-7 *PRE: 1.6 kb of the *Fab-7 *PRE flanking the *mw *reporter; (bottom row) *vg *PRE: 1.6 kb of the *vg *PRE flanking the *mw *reporter. **(B) **Quantification of eye pigment in heads of flies shown in (A). Pigment levels are expressed as percentage of wild-type pigment. 50 heads per assay were used, of an equal mix of male and female flies, aged 5 days. Mean and standard deviation of two biological replicates are shown. **(C) **Repression index w calculated from mean pigment levels in (B) as no PRE/PRE for each transgenic line at each landing site for heterozygotes and homozygotes. The graph shows the maximum, minimum and mean (horizontal line) repression index at four landing sites for each PRE, in (left) heterozygotes and (right) homozygotes. Values <1 indicate activation. **(D) **Pairing-sensitive silencing (PSS) was calculated from mean pigment levels in (B) as heterozygote/homozygote for each transgenic line, normalized to the same ratio calculated for the 'no PRE' control line at the same landing site. Values >1 (horizontal dotted line) indicate PSS.

**Figure 4:**
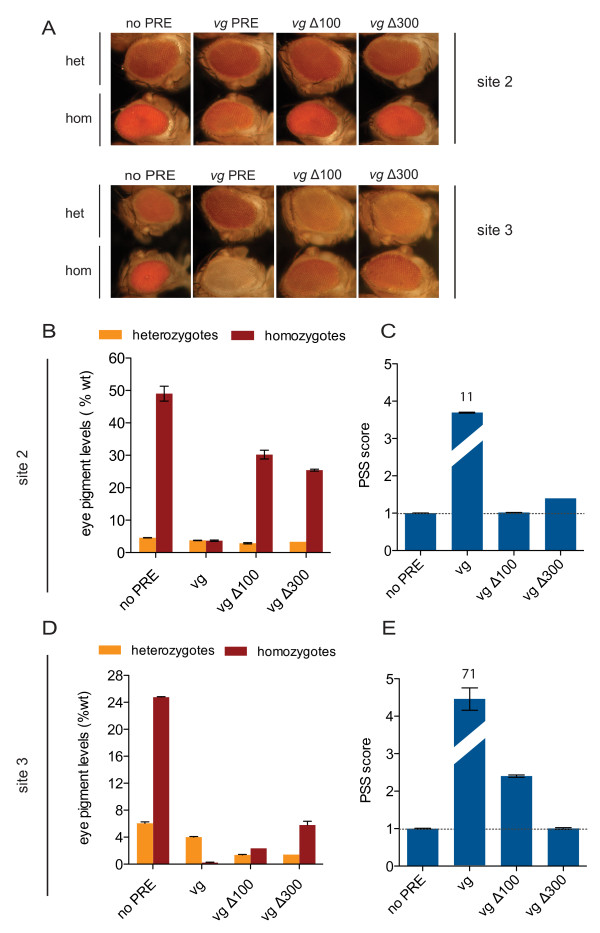
**Deletion analysis of the *vg *Polycomb response element (PRE)**. **(A) **Eye phenotype of transgenic fly lines; 5-day-old male flies are shown. In each row, (top) heterozygous and (bottom) homozygous transgenic flies are shown, carrying the *mw *reporter alone (no PRE), the 1.6 kb *vg *PRE, a deletion of the first 106 bp (*vg*Δ100) and the first 291 bp (*vg*Δ300) of the 1.6 kb *vg *PRE, at landing sites (top) 2 and (bottom) 3. **(B,D) **Quantification of pigment levels of the heterozygous and homozygous transgenic *vg*Δ100 and *vg*Δ300 deletion lines at landing sites **(B) **2 and **(D) **3. **(C,E) **Pairing-sensitive silencing (PSS) of transgenic *vg*Δ100 and *vg*Δ300 deletion lines at landing sites **(C) **2 and **(E) **4, calculated as in Figure 3.

To test whether this 1.6 kb PRE fragment is also able to accomplish Polycomb-dependent silencing, we crossed transgenic flies carrying pKC27_*mw *either with or without the 1.6 kb *vg *PRE at landing sites 1, 2 or 3 (Table 1) into a *Polycomb *(*Pc*) mutant background (Figure 2D). In the wild-type background, addition of the PRE to the *mw *reporter caused repression of *mw *at all three landing sites (Figure 3D, top row, Figure 4). Control flies (no PRE), heterozygous or homozygous for the *mw *reporter, showed no change in a *Pc*^*XL5 *^mutant background at all three sites (Figure 2D and data not shown). Likewise, flies heterozygous for the *vg *PRE showed no detectable change in the *Pc*^*XL5 *^mutant background (data not shown). By contrast, flies homozygous for the *vg *PRE showed a loss of silencing at all three sites in the *Pc*^*XL5 *^mutant background, indicated by an increase in eye pigmentation (Figure 2D, bottom row). In summary, these results show that the 1.6 kb *vg *PRE functions as a Polycomb-dependent silencer.

### The *vg *and *Fab-7 *PREs show site-specific differences in silencing behavior

To determine whether the *vg *and *Fab-7 *PREs have inherently different functions, we used the ΦC31 site-specific integration tool to perform a quantitative comparison of the 1.6 kb *vg *and *Fab-7 *PREs. To distinguish between the effects of genomic environment and the inherent properties of the PRE sequence, we compared the PREs to control constructs lacking the PRE at each of the four landing sites (Table 1). To obtain a quantitative readout of *mw *expression levels, we extracted and measured the eye pigment from the heads of 5-day-old flies for each transgenic line, from flies both heterozygous and homozygous for the transgene (Figure 3).

To determine the effect of the genomic environment on the level of *mw *activity in the absence of the PRE, we first examined the control lines at each landing site (Figure 3A,B (no PRE)). Surprisingly, three of the four transgenic lines had strong pairing-sensitive activation (PSA) of *mw *in the absence of the PRE. The expected expression level of the *mw *reporter gene was twofold lower in heterozygotes (carrying one copy of the *mw *gene) compared with homozygotes (carrying two copies of the *mw *gene). This was seen at site 4, where heterozygotes had 7% and homozygotes 13% of wild-type pigment levels (Figure 3B). However, at sites 1, 2 and 3, the homozygous pigment levels were four to 12 times higher in heterozygotes (Figure 3B). This suggests a pairing-dependent activation of the *mw *reporter at sites 1, 2 and 3, and gives a quantitative measure of the extent to which the genomic environment of the landing sites affects *mw *reporter-gene activity in the absence of a PRE.

We next quantified the effect of each PRE on the *mw *levels at each landing site (Figure 3B). This analysis revealed PRE- specific effects at each site. For example, in both heterozygotes and homozygotes at sites 1 and 2, the *Fab-7 *PRE repressed *mw *more strongly than the *vg *PRE, but at site 4, the reverse was true. Indeed, *Fab-7 *gave higher levels of *mw *than the control line at site 4, indicating that this element acts as an activator at this site. Remarkably, at site 3, opposite effects were observed for the two PREs in heterozygotes and homozygotes, with the *vg *PRE giving weaker repression than the *Fab-7 *PRE in heterozygotes, but stronger repression in homozygotes. These site-dependent differences in silencing strength clearly indicate that the two PREs interact differently with the genomic environment of each landing site.

These observed site-specific differences in PRE behavior raised the question of whether the *Fab-7 *and *vg *PREs have intrinsic properties that are inherent to each PRE despite the effect of genomic environment. To address this question, we calculated a 'repression index' for each PRE, and used this to compare their general behavior in heterozygotes and homozygotes (Figure 3C). The repression index is defined as the ratio of 'no PRE' to 'PRE' pigment levels at each site, thus a higher repression index indicates stronger silencing. Figure 3C shows the minimum, maximum and mean repression index for all four sites for each PRE in heterozygotes and homozygotes (values <1 indicate activation). Surprisingly, this analysis revealed striking differences in the properties of the *Fab-7 *and *vg *PREs in heterozygotes and homozygotes. In heterozygotes (Figure 3C, left panel), the effects of *Fab-7 *PRE on *mw *differed strongly from site to site, ranging from a 3.5-fold repression to a 1.5-fold activation. The wide range of values for *Fab-7 *in heterozygotes indicates high sensitivity of *Fab-7 *PRE to the landing-site environment, and an ability to silence strongly at some sites but to activate at one site. By contrast, the narrow range of values for the *vg *PRE in heterozygotes, with a mean at approximately 1.5-fold repression (Figure 3C, left panel) indicates that this PRE was less sensitive to its genomic environment than *Fab-7*, giving mild silencing of *mw *at all sites tested. (Figure 3C, left panel).

In homozygotes, this tendency was reversed (Figure 3C, right panel). Although the silencing properties of both PREs were typically stronger in homozygotes than in heterozygotes, the *Fab-7 *PRE showed less repression on average, ranging from 18-fold repression to 1.2-fold activation, compared with the *vg *PRE, which ranged from 49-fold to 2.3-fold repression. Furthermore, the *vg *PRE had more site-dependent variation than *Fab-7*, indicating a higher sensitivity to the genomic environment at the four sites tested (Figure 3C, right panel). In summary, this analysis indicates that the *vg *and *Fab-7 *PREs do indeed have inherently different properties in terms of their ability to repress the *mw *reporter in the heterozygote and homozygote states, and their sensitivity to genomic environment in each of these states.

To further evaluate differences in PRE behavior, we examined PSS, a phenomenon typical of *Drosophila *PREs [19,20]. In PSS, the reporter gene is more strongly repressed in flies homozygous for a PRE transgene than in flies heterozygous for the same PRE transgene. PSS is thought to be caused by PcG proteins forming higher-order complexes on paired PREs. We used the eye-pigment assay data to define a quantitative 'PSS score', by calculating the ratio of heterozygote to homozygote eye pigment for each PRE line, and normalizing these values to the same ratio calculated for the 'no PRE' control at the same landing site, for which we assumed no PSS. Thus, a PSS score close to 1 indicates that the ratio of homozygous to heterozygous eye pigment is similar to that of the 'no PRE' line, and thus that there is no or only very weak PSS (Figure 3D); for example, this was the case for *Fab-7 *at sites 3 and 4 and *vg *at site 4 (Figure 3D). By contrast, we found strong PSS for both PREs at sites 1 and 2. Intriguingly, these site-specific PSS scores agree well with the PSA levels we found for the 'no PRE' construct, suggesting that the inherent pairing properties of each locus may influence the extent to which PRE-dependent PSS occurs. However, we also found a striking difference between the *Fab-7 *and *vg *PREs at site 3. Whereas the *Fab-7 *PRE had a PSS score of 1.1 at this site,(indicating no PSS) the *vg *PRE had a PSS score of 71 (Figure 3D) with homozygous flies displaying completely white eyes (Figure 3A).

Taken together, these results indicate quantitative differences in the behavior of the *vg *and *Fab-7 *PREs in terms of reporter-gene repression and PSS, which reflect both specific interactions of each PRE with the genomic environment at each landing site, and the inherent properties of each PRE.

### Deletion analysis of the *vg *PRE identifies sequences essential for PRE function

A set of motifs consisting of the ZESTE, GAF/PSQ and PHO/PHOL binding sites are necessary, but not sufficient for PRE function [37]. Sequence mining has revealed a novel motif frequently found in predicted PREs, termed the GTGT motif [8]. This motif has also been found independently to be enriched in genomewide ChIP datasets [11], but has not been tested experimentally. The 1.6 kb *vg *PRE is highly enriched in this motif (Figure 2C) To investigate whether GTGT repeats play a role in PRE function, we generated deletion constructs lacking sequences containing these motifs (Figure 2C). The *vg*Δ100 construct lacks the first 106 bp, containing the first block of GTGT repeats, and *vg*Δ300 lacks the first 291 bp of the 1.6 kb *vg *PRE, containing the first and second blocks of GTGT repeats. As the *vg *PRE had the strongest PSS at sites 2 and 3 (Figure 3D), the deletion constructs were integrated at these sites. The effects of deletions were evaluated by pigment assay and PSS score as described above.

At site 2, at which the 1.6 kb *vg *PRE caused partial repression of *mw *in homozygotes, the *vg*Δ100 deletion line had little effect on reporter-gene activity in heterozygotes, but a pronounced loss of silencing in homozygotes (Figure 4B) and a complete loss of PSS (Figure 4C). These effects were not increased by further deletion in the *vg*Δ300 constructs (Figure 4B,C). Similarly, at site 3, at which the 1.6 kb *vg *PRE caused complete repression of *mw *in homozygotes, the *vg*Δ100 homozygotes had partial loss of silencing and PSS, whereas the *vg*Δ300 homozygotes had a more pronounced loss of silencing and total loss of PSS (Figure 4A,D,E). Remarkably, in heterozygotes at site 3, both the *vg*Δ100 and *vg*Δ300 deletion lines had an increase in silencing compared with the 1.6 kb *vg *PRE (Figure 4A,D). In summary, by comparing the same deletion constructs at two different landing sites, this analysis distinguished between landing site-specific effects of the deletions such as those seen in heterozygotes at site 3, and effects that are intrinsic to the PRE sequences themselves, such those seen in homozygotes at both sites. The fact that the deletion of the first 106 bp leads to loss of repression in homozygotes at both sites demonstrates that this sequence is essential for PRE function.

### The GTGT motif is essential for *vg *PRE silencing

**Figure 5:**
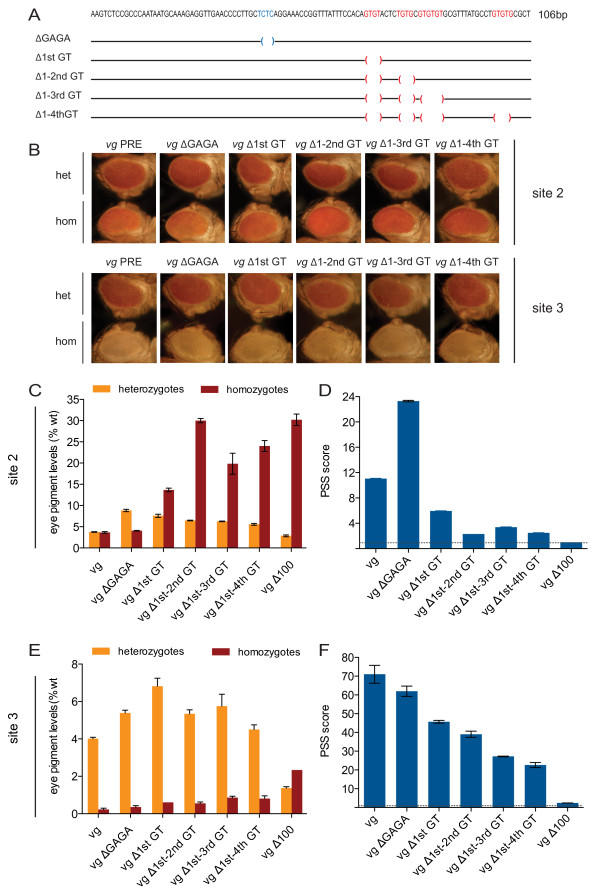
**Mutational analysis of the *vg *Polycomb response element (PRE)**. **(A) **Top: sequence of the first 106 bp of the 1.6 kb *vg *PRE containing one GAGA site (blue) and four GTGT repeats (red). Bottom: schematic view of deletion constructs for the GAGA site (*vg*ΔGAGA), the first GTGT repeat (*vg*Δ1^st ^GT), the first and second GTGT repeats (*vg*Δ1^st^-2^nd ^GT), the first to third GTGT repeats (*vg*Δ1^st^-3^rd ^GT) and the first to fourth GTGT repeats (*vg*Δ1^st^-4^th ^GT) within the first 106 bp of the 1.6 kb *vg *PRE are shown. **(B) **Eye phenotypes of heterozygous and homozygous transgenic *vg*ΔGAGA and *vg*ΔGT deletion lines at landing sites (top) 2 and (bottom) 3; 5 day-old male flies are shown. **(C,E) **Quantification of pigment levels of heterozygous and homozygous transgenic *vg*ΔGAGA and *vg*ΔGT deletion lines at landing sites **(C) **2 and **(E) **3. The *vg*Δ100 pigment levels from Figure 4 are shown for comparison. **(D**,**F) **Pairing-sensitive silencing (PSS) of the transgenic *vg*ΔGAGA and *vg*ΔGT deletion lines at landing sites **(D) **2 and **(F) **3, calculated as in Figure 3.

The *vg*Δ100 deletion removed several GTGT repeats and a single GAGA motif (Figure 2C, Figure 5A). To gain further insight into the role of this GAGA site and the GTGT repeats in PRE silencing function, we used site-directed mutagenesis to generate *vg *PRE constructs with deletions of single motifs or groups of motifs (Figure 5A) within the first 106 bp of the 1.6 kb *vg *PRE. All constructs were integrated at sites 2 and 3.

We first addressed the role of the GAGA motif (Figure 5A). The GAF protein or GAGA binding motifs have been reported to be required for activation or silencing, or to be dispensable at different PREs [57]. In heterozygotes, *vg*ΔGAGA lines in both sites 2 and 3 produced a loss of silencing of 1.4-fold to twofold compared with the 1.6 kb *vg *PRE line. However, deletion of the GAGA site had no detectable effect on the PRE silencing function in homozygotes at either site (Figure 5B-E), thus the GAGA site cannot account for the pronounced loss of silencing seen in homozygotes of the *vg*Δ100 deletion lines (Figures 4, Figure 5C,E).

We next addressed the role of the GTGT repeats. Deletion of the first GTGT repeat (*vg*Δ1^st ^GT) at site 2 resulted in a substantial loss of silencing in both heterozygotes (twofold) and homozygotes (threefold), resulting in a twofold reduction in PSS (Figure 5B-D). Replacement of the GTGT sequence with a different sequence led to similar loss of silencing, indicating a specific requirement for the GTGT motif (not shown). Strikingly, deletion of the first and the second GTGT repeat (*vg*Δ1^st^-2^nd ^GT) had no further effect in heterozygotes but a dramatic (7.5-fold) loss of silencing in homozygotes, similar to that seen with the Δ100 deletion (Figure 5B-D). Deletion of further GTGT repeats did not lead to further loss of silencing, thus this analysis identified an essential contribution of the first two GTGT repeats at this site. These observations were further reflected in PSS levels (Figure 5D).

To evaluate whether the loss of silencing seen upon deletion of GTGT repeats reflects a PRE-specific requirement, or rather an interaction of these motifs with the genomic environment of the landing site, we analyzed the same series of deletion constructs at site 3. Remarkably, despite the strong silencing in the homozygous 1.6 kb PRE line at site 3, the trend for the GTGT deletion series was similar to that seen for site 2 (Figure 5B, E, S2). At site 3, all GTGT repeat deletion lines had 1.2-fold to 1.5-fold higher pigment levels in heterozygotes than in the 1.6 kb *vg *PRE (Figure 5B,E). Homozygous pigment levels increased 2.4-fold upon deletion of the first or first and second GTGT. In contrast to site 2, additional deletion of the third GTGT repeat resulted in a further loss of silencing of fourfold (Figure 5E; see Additional file 2, Figure S2). Additional deletion of the fourth GTGT repeat had no further effect on silencing (Figure 5E). These observations were also reflected in the PSS score, with successive deletion of the GTGT repeats resulting in a gradual decrease in PSS (Figure 5F). Thus, although the *vg *PRE acts as a strong silencer at site 3, this repression is partly relieved by deletion of the GTGT motifs. Taken together, these results show that the GTGT motif plays an essential role in *vg *PRE silencing function at both genomic loci tested.

## Discussion

Classic *Drosophila *PRE assays have used random integration by P-element insertion. However PREs are extremely sensitive to genomic position, thus comparisons between different elements and evaluation of the effect of mutations, has previously relied upon average behavior of multiple different transgenic lines [37,43]. In this study, we used the ΦC31 site-specific integration tool to compare different PRE elements, mutated variants and control constructs at identical genomic locations, enabling quantitative comparisons.

### Inherent properties of *Fab-7 *and *vg *PREs

In addition to differences in their interaction with the landing-site environment (discussed below), the quantitative analysis presented here revealed surprising differences in the inherent properties of the two PREs, in terms of their influence on reporter genes in the heterozygote and homozygote states (Figure 3C). Whereas the *Fab-7 *PRE had a wide range of reporter-gene expression levels at the four sites in heterozygotes and a more uniform output in homozygotes, this trend was reversed for the *vg *PRE, which gave a uniform mild silencing at all sites in heterozygotes, in contrast to the wide site-to-site differences in levels of reporter-gene activity in homozygotes. The data in heterozygotes suggest that the *Fab-7 *PRE may be more sensitive to *cis *effects whereas, the *vg *PRE seems to buffer against them. This demonstration of different PRE properties is consistent with several studies showing differences in PRE behavior [24,42-44]. It would be of great interest in future to use the site-specific quantitative approach described here for the systematic comparison of other PREs.

We quantified the extent to which each PRE mediates PSS at each site. Interestingly, both the BX-C and the endogenous *vg *locus have been shown to be subject to transvection, in which the expression of a gene can be affected by its homologous counterpart in *trans *[58,59]. In the case of the BX-C, several PREs, including the *Fab-7 *PRE [60] have been shown to be able to mediate *trans *effects, but the transvection observed at the endogenous *vg *locus [59] has not been attributed to specific sequences. The strong PSS we found for the *vg *PRE at sites 1, 2 and 3 suggests that this element may play a role in the transvection properties of the *vg *locus.

The quantitative analysis presented here identified important differences between the *Fab-7 *and *vg *PREs. The molecular mechanisms that underlie these differences will be a key question for future studies. A recent study in which core sequences of Hox PREs were exchanged at the same genomic location has shown that these sequences can effectively substitute for each other [40], raising the possibility that Hox PREs are rather similar, and that the differences we found could be due to the comparison of Hox and a non-Hox PRE. However, in experiments with a 1.6 kb fragment of the Hox *bxd *PRE, we found different levels of silencing to *Fab-7 *at all sites tested, and were able to recover transgenes at some sites only by PCR screening, owing to strong silencing in heterozygotes (data not shown). Thus, we propose that the difference between *Fab-7 *and *vg *PREs that we found lies less in a fundamental difference between Hox and non-Hox PREs than in differences between the sequences flanking the core region.

### Site-specific differences between *Fab-7 *and *vg *PREs

**Figure 6:**
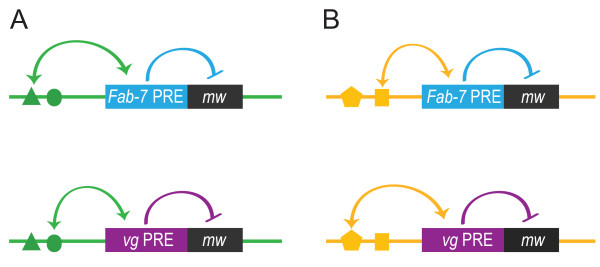
**Polycomb response elements (PREs) show both site-specific and inherent behavior**. The *Fab-7 *(blue) and *vg *(purple) PREs are shown at two different landing sites **(A,B)**. Each PRE has inherent properties in repressing the *mw *reporter (blue and purple arrows). We propose that each PRE also interacts specifically with different enhancers or repressors at each landing site (green and yellow arrows; enhancers/repressors shown as solid symbols). The output of the *mw *promoter depends on the properties of the PRE, on its interaction with the landing-site environment, and on whether the PRE is heterozygous or homozygous (see main text for details).

Genomic position effects on PRE behavior have been found in studies using random P-element insertion of PRE reporter transgenes [4,5,19]. However, it has not been clear whether these effects are due to the general transcriptional activity at a given site or whether each PRE interacts specifically with local genomic features. Our data strongly support the latter interpretation, because the *Fab-7 *and *vg *PREs showed strikingly different behaviors at each site. These differences were most pronounced at site 3, at which the *Fab-7 *PRE showed no evidence of no PSS whereas the *vg *PRE had a PSS score of 71 (71-fold PSS) (Figure 3D), and at site 4, at which the *Fab-7 *PRE induced activation, whereas the *vg *PRE induced repression of the reporter gene (Figure 3B). These results clearly indicate PRE-specific interactions with the genomic environment at each site, and suggest that sequences within each PRE may be interacting with a selected set of enhancers and/or repressors (Figure 6).

This interpretation is consistent with a recent study of an *engrailed *PRE reporter inserted at different sites by random P-element insertion [61], showing that the reporter gene reflects patterns of nearby enhancers in a PRE-dependent manner. Our work provides a further example of selective interaction of PREs with their genomic environment, and raises the intriguing possibility that different PREs may interact preferentially with different regulatory elements (Figure 6). We have focused here on the quantitative analysis enabled by the *mw *reporter combined with the pigment assay, thus limiting our study to the readout in the adult eye. However, it would also be of great interest to determine whether the *Fab-7 *and *vg *reporters are expressed in distinct patterns during development at each landing site, as this would reveal whether the quantitative differences between the two PREs that we observed indeed reflect specific interactions with different regulatory elements at each landing site. As our constructs did not contain a *LacZ *reporter (similar to those reported previously [61]), this analysis would require *in situ *hybridization to the *mw *cDNA.

### Inherent properties of landing sites

Despite PRE-specific differences in behavior, some characteristics of each landing sites did seem to be reflected by both PREs. For example, we found that at sites 1, 2 and 4, the extent of PSS shown by each PRE closely mirrored the extent of PSA of the reporter gene lacking the PRE (Figure 3D). This observation emphasizes the advantage of examining control constructs and PRE constructs at identical locations, enabling a quantitative evaluation of the contribution of both the locus itself and the PRE sequences to pairing-dependent effects. At sites 1 and 2, we found strong PSA and strong PSS by both PREs, whereas at site 4, we found no PSA and little or no PSS by either PRE. Pairing of homologous chromosomes occurs in somatic cells in *Drosophila*, and can facilitate both activation and repression of genes [62,63]. It has been proposed that PSS, commonly observed for PRE transgenes, is due to the interactive properties of PcG complexes, thereby facilitating pairing of two PREs on homologous chromosomes [19,63]. Such interactions have also been proposed to account for long-range contacts between PREs at non-homologous sites. However, it has recently been found for the *Fab-7 *PRE that these long-distance interactions depend not on the PRE but on the adjacent 1257 bp insulator, 778 bp of which is contained in the 1.6 kb *Fab-7 *fragment studied here (see Additional file 1, Figure S1) [64]. It remains to be seen whether the *vg *PRE also contains an insulator element. Our data for sites 1, 2 and 4 suggest that the inherent pairing properties of a locus may also contribute to the strength of PRE or insulator-mediated PSS. In the future, it will be very informative to use the approach described here to quantify the relative contributions of PRE sequences, insulator sequences and genomic environment to PSS using comparison of different PRE deletion constructs at these defined sites. Indeed, the distinct behavior of the two PREs at site 3, at which *Fab-7 *produced no PSS and *vg *produced 71 fold PSS, demonstrates that the extent of somatic pairing of a region is not alone sufficient to determine PSS.

The only site at which neither PRE gave strong PSS was site 4. Notably, this site is telomeric (Table 1), and telomeric regions have been shown to be able to recruit PcG and TrxG proteins and to induce PSS [65], thus it is surprising that no PSS was observed at this site. However, the previous study also reported that a specific subset of PcG and TrxG proteins functions at telomeres. As the endogenous locations of *Fab-7 *and *vg *PREs are not at telomeres, these PREs may lack telomere-specific sequences required for correct PRE function at this location.

Furthermore, it is interesting to note that each landing site is situated in a specific chromatin type, as defined previously [66] (Table 1), which may in part account for the differences in PRE behavior seen at each site. For example, site 4, at which neither PRE showed strong silencing or PSS, is located close to 'yellow' chromatin containing active euchromatin (Table 1). Thus it is possible that, despite the telomeric location of site 4, the adjacent active-chromatin environment affects the behavior of both PREs. By contrast, site 2 is located in 'red'chromatin, also containing active euchromatin but defined by a different set of binding factors. Here, the *vg *PRE was able to repress the *mw *reporter, but not as strongly as at site 3, which is located in 'blue' chromatin, characterized by PcG protein binding and enrichment of the repressive chromatin marker H3K27me3. Interestingly, comparison of the deletion lines at these two sites showed that the *vg *PRE was more sensitive to changes in DNA sequence at site 2 than at site 3, which could be explained by the difference in chromatin type, with site 3 more strongly reinforcing *vg *PRE silencing function because of its repressive chromatin environment. However, caution must be exercised in this interpretation, as the *Fab-7 *PRE showed weaker silencing and PSS at site 3 than at site 2, suggesting that for the *Fab-7 *PRE, some intrinsic sequence property mediates local interactions that overcome the inherently repressive nature of the chromatin environment at site 3.

A search for local sequence features that might account for site-specific differences in PRE behavior revealed different numbers of the motifs GTGTG, GAGAG (potential GAF binding sites) and GCCAT (potential PHO binding sites) (Table 1). However, no clear correlation emerged from this analysis, again suggesting that additional sequences present at each landing site interact specifically with each PRE.

### *vg *PRE deletions and the GTGT motif

Deletion of the first 106 bp of the *vg *PRE led to loss of repression in homozygotes at the two sites tested, indicating that this sequence is essential for PRE function. Deletion of single motifs within this sequence showed that small deletions gave a detectable change in silencing properties. The quantitative changes in PRE output upon deletion of the 106 bp sequence and of the short motifs were within the same range as the differences detected for a PRE of identical sequence inserted at different sites in the genome. This emphasizes the advantages of the site-specific approach taken here, as analysis of these deletions and mutations by a random integration approach would have been difficult to interpret.

This 106 bp sequence contains a single GAGAG motif and several GTGT motifs. GAGAG sites can be bound by the GAF [33] and PSQ [34,35] proteins. GAGAG sites or the GAF protein have been shown in different studies to have roles in both activation and silencing at PREs, or to have no effect on PRE function [57]. The GTGT motif has been identified in PREs by two independent sequence-mining approaches [8,11], but has not previously been tested experimentally.

Deletion of the single GAGAG motif resulted in a marked loss of silencing in heterozygotes at both sites, thus this site seems to be involved in silencing at the *vg *PRE, and may thus be a target for the PcG protein PSQ [34,35] or may be important for cooperative binding of GAF or PSQ with PHO, as shown for the *bxd *PRE [40]. However, because the deletion of the GAGA site had no effect in homozygotes, it cannot account for the marked loss of silencing we observed in homozygotes of the 106 bp deletion lines. Thus we reason that additional sequences within this region must be required for silencing.

Site-specific sequential mutation of four GTGT motifs within this 106 bp region resulted in pronounced loss of silencing in homozygotes at both sites tested, identifying an essential role for this motif in silencing at the *vg *PRE. Future studies will aim to elucidate the molecular mechanism by which this motif acts on *vg *PRE silencing. Proteins that bind specifically to this sequence have not been identified to date [67]. Bioinformatic analysis of PREs that contain the GTGT motif [8] did not identify any clear correlation between the occurrence of the motif in a PRE and the class of genes with which it is associated (data not shown). Likewise, in another study [11], the authors did not report a correlation between the occurrence of this motif and a particular class of genes, but rather detected the GTGT motif to be specifically enriched in regions with a particular chromatin 'anatomy', namely, those regions enriched for PRC1 proteins and with H3K4Me3 at the transcription start site. The motif was less enriched at transcription start sites with low H3K4Me3 enrichment, thus the GTGT motif may distinguish between different promoter architectures.

We report elsewhere that both the endogenous *vg *PRE and the transgenic *vg *PRE constructs are transcribed into non-coding RNA (Lempradl *et al.*, submitted) thus the effect of the GTGT motif on silencing the *mw *reporter in the adult eye may be mediated via transcriptional or post-transcriptional effects on non-coding RNA.

Finally, it is possible that the motif plays an indirect role in silencing, for example by affecting nucleosome positioning [52,68] or by insulating the PRE from the effects of nearby chromatin [52]. Ultimately it will be essential to test the role of the 106 bp region and the GTGT motifs within it at the endogenous *vg *locus, using homologous recombination [69]. However this technique is time-consuming and does not lend itself readily to analysis of large numbers of constructs.

## Conclusions

The ΦC31 mediated site-specific integration approach described here demonstrates the power and sensitivity of the technique for the rapid and quantitative analysis of several PREs and multiple PRE sequence variants, giving a number of unexpected insights into PRE function. This analysis revealed that the *Fab-7 *and *vg *PREs have fundamentally different properties, both in terms of their interaction with the genomic environment and their inherent silencing abilities. Furthermore, we used the ΦC31 tool to examine the effect of deletions and mutations in the *vg *PRE, identifying a 106 bp region containing a previously predicted motif, and demonstrating an essential role for this motif in silencing. Using quantitative analysis of mutated *vg *PRE variants at identical genomic sites, we found that changes in as few as four base pairs have profound effects on PRE function, thus illustrating the sensitivity of site-directed integration as a tool for the quantitative dissection of elements of PRE design.

## Methods

### Transgenic constructs, cloning

The pKC27_*mw *vector was generated from pKC27 (Kuan-chung Su and Barry Dickson, unpublished, available on request) by replacing an *Ade*I-*Xba*I fragment containing the *white *promoter, with a 402 bp *Ade*I-*Xba*I fragment of pCaSpeR4 containing the *miniwhite *promoter [70] (flybase: FBtp0000155). A modified version of pCaSpeR4 was used for this, from which the 42 bp between the *Eco*RI and *Spe*I sites in the polylinker had been removed by *Eco*RI/*Spe*I digestion, end-filling and re-ligation, thus removing several cloning sites that were present in pKC27. The transgenic PRE constructs of the *Fab-7 *and *vg *PRE were obtained by PCR amplification on genomic DNA using the primers shown in Table 2. PREs, variants and control constructs were cloned with *Not*I/*Xba*I or *Not*I/*Spe*I into the pKC27_*mw *vector.

**Table 2:** Primers used in this study.

Primer	Direction	Sequence (5'→3')	Enzyme site
*Primers for transgenic constructs*			

*vg1.6 kb*	Forward	**TAAAGCGGCCGC**AAGTCTCCGCCCAATAAT	*NotI*

*vg*Δ100	Forward	**TAAAGCGGCCGC**AGTTTGTGTGAGAGTGAGC	*NotI*

*vg*Δ300	Forward	**TAAAGCGGCCGC**CGTAATTAAAACCGAAGG	*NotI*

*vg*1.6 kb, *vg*Δ100, *vg*Δ300	All reverse	**GCGCTTTCTA**GAGAGCATATAGAAGTGGTCGAA	*XbaI*

*Fab-7*	Forward	**TAAAGCGGCCGC**GGAATTGTGTGGACGATG	*NotI*

*Fab-7*	Reverse	GGCGCTTACTAGTGCACAGAGAGTGCAGAAAG'	*SpeI*

*Primers for mutagenesis*			

*vg*ΔGAGA	Forward	AAGAGGTTGAACCCCTTGAGGAAACCGGTTTATTTC	-

*vg*ΔGAGA	Reverse	GAAATAAACCGGTTTCCTCAAGGGGTTCAACCTCTT	-

*vg*Δ1^st^GT	Forward	CAGGAAACCGGTTTATTTCCACAACTCTGTGCGTG	-

*vg*Δ1^st^GT	Reverse	CACGCACAGAGTTGTGGAAATAAACCGGTTTCCTG	-

*vg*Δ1^st^-2^nd^GT	Forward	GGTTTATTTCCACAACTCCGTGTGTGCGTTTATGCC	-

*vg*Δ1^st^-2^nd^GT	Reverse	GGCATAAACGCACACACGGAGTTGTGGAAATAAACC	-

*vg*Δ1^st^-3^rd^GT	Forward	*GT*TTATTTCCACAACTCCCGTTTATGCCTGTGTGCG	-

*vg*Δ1^st^-3^rd^GT	Reverse	CGCACACAGGCATAAACGGGAGTTGTGGAAATAAAC	-

*vg*Δ1^st^-4^th^GT	Forward	CAACTCCCGTTTATGCCCGCTAGTTTGTGTGAGA	-

*vg*Δ1^st^-4^th^GT	Reverse	TCTCACACAAACTAGCGGGCATAAACGGGAGTTG	-

Bold sequences refer to primer tags that contain a restriction site (named in following column).

### Site-directed mutagenesis

Site-directed mutagenesis of PRE motifs was performed on pKC27_*mw *carrying the 1.6 kb *vg *PRE (QuikChange^® ^II Site-Directed Mutagenesis Kit; Stratagene, La Jolla, CA, USA) according to the manufacturers' protocol using the primers shown in Table 2.

### Fly stocks and crosses

Transgenic fly lines were obtained as described [71] by co-injection of the pKC27_*mw *plasmid carrying the construct of interest with the helper plasmid pKC40 (Kuan-chung Su and Barry Dickson, unpublished, available on request) encoding ΦC31 integrase into landing-site embryos. The following landing-site lines (details in Table 1) were used: sites 1 to 3: *yw; p[w3', y+, attP]; +.*site 4: *yw; +; p[w3', y+, attP]*. Crosses to *Pc*^*XL5 *^mutants were performed as described previously [36] for PRE and control lines at sites 1 to 3, in which the landing site is on chromosome II, enabling the homozygous PRE transgene to be combined with the heterozygous *Pc*^*XL5 *^mutation on chromosome III.

### Phenotypic analysis of flies

Eye-pigment quantification was performed according to [72] with minor adaptations: 30-50 heads instead of 10 were used per assay to achieve robust measurements for a range of eye-pigment levels. Transgenic flies were photographed with a microscope (Lumar V12 Stereo Microscope; Carl Zeiss GmbH, Vienna, Austria) at 65× magnification, 2 seconds exposure, using Insight SPOT software (Diagnostic Instruments, Inc, Sterling Heights, MI, USA).

## Competing interests

The authors declare that they have no competing interests.

## Authors' contributions

HO performed the pigment assays, designed and generated constructs and transgenic lines for the *vg *deletion and mutation constructs, and wrote the manuscript jointly with LR. BD generated constructs and transgenic lines for full-length *Fab-7*, *vg *and control constructs. SB generated and characterized the landing-site lines. LR supervised HO and BD, conceived and designed the study, and wrote the manuscript jointly with HO. All authors read and approved the final manuscript.

## Supplementary Material

Additional file 1 **Figure S1. *Fab-7 *PRE motifs**. DNA motifs in the 1.6 kb *Fab-7 *PRE fragment used in this study. The grey box corresponds to the highest-scoring region of the Polycomb response element (PRE) [8], and contains the minimal 219 bp PRE core sequence previously identified [56] (black line below diagram). Red line below plot indicates position of the Fab-7 insulator sequences contained in the 1.6 kb PRE [64]. ZESTE, GAGA factor/Pipsqueak (GAF/PSQ) and Pleiohomeotic/Pleihomeotic-like (PHO/PHOL) DNA-binding motifs are shown. In addition, the GTGTG motif, found to be enriched in many PREs [8,11], is enriched in the sequences flanking the core region. Click here for file

Additional file 2 **Figure S2. Eye color comparison of intact and mutated *vg *PREs at site 3; 5-day-old male flies are shown**. (Top) A *w*^*1118 *^mutant; (bottom left) homozygous 1.6 kb *vg *PRE at site 3; (bottom right) homozygous 1.6 kb *vg*Δ1^st^-3^rd ^GT at site 3. The 1.6 kb *vg *PRE line has essentially identical eye color to that of the *w*^*1118 *^mutant, whereas the deletion of the first three GT repeats leads to a visible increase in eye pigmentation. Click here for file
