# Supplementary figures and images for: Quantitative analysis of polycomb response elements (PREs) at identical genomic locations distinguishes contributions of PRE sequence and genomic environment
Source: Epigenetics Chromatin. 2011 Mar 16;4:4. doi: 10.1186/1756-8935-4-4 (PMC3070613; doi:10.1186/1756-8935-4-4)

# *Fab-7* PRE

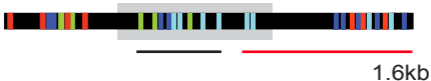

|   |          |        |
|---|----------|--------|
| ■ | GAF/Psq  | GAGAG  |
| ■ | Zeste    | YGAGYG |
| ■ | Pho/Phol | GCCAT  |
| ■ | GT       | GTGT   |

Supplement: Additional file 1 — Figure S1. Fab-7 PRE motifs. DNA motifs in the 1.6 kb Fab-7 PRE fragment used in this study. The grey box corresponds to the highest-scoring region of the Polycomb response element (PRE) [8], and contains the minimal 219 bp PRE core sequence previously identified [56] (black line below diagram). Red line below plot indicates position of the Fab-7 insulator sequences contained in the 1.6 kb PRE [64]. ZESTE, GAGA factor/Pipsqueak (GAF/PSQ) and Pleiohomeotic/Pleihomeotic-like (PHO/PHOL) DNA-binding motifs are shown. In addition, the GTGTG motif, found to be enriched in many PREs [8,11], is enriched in the sequences flanking the core region. [file 1756-8935-4-4-S1.PDF]

*w1118*

*vg* PRE

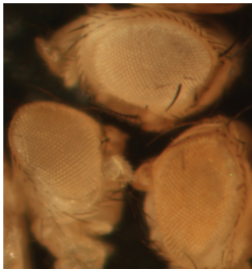

$\Delta 1$ -3rd GT

Supplement: Additional file 2 — Figure S2. Eye color comparison of intact and mutated vg PREs at site 3; 5-day-old male flies are shown. (Top) A w1118 mutant; (bottom left) homozygous 1.6 kb vg PRE at site 3; (bottom right) homozygous 1.6 kb vgΔ1st-3rd GT at site 3. The 1.6 kb vg PRE line has essentially identical eye color to that of the w1118 mutant, whereas the deletion of the first three GT repeats leads to a visible increase in eye pigmentation. [file 1756-8935-4-4-S2.PDF]
